# Hepatitis B vaccination coverage amongst healthcare workers in a tertiary academic hospital in Gauteng province, South Africa

**DOI:** 10.4102/sajid.v37i1.393

**Published:** 2022-07-27

**Authors:** Lufuno L. Razwiedani, Ntlogeleng M. Mogale, Muthuhadini P.B. Mawela

**Affiliations:** 1 Department of Public Health Medicine, Faculty of Health Sciences, Sefako Makgatho Health Science University, Pretoria, South Africa; 2 Department of Public Health, Faculty of Health Sciences, Sefako Makgatho Health Science University, Pretoria, South Africa; 3 Department of Pediatrics, Faculty of Health Sciences, School of Medicine, Sefako Makgatho Health Sciences University, Pretoria, South Africa

**Keywords:** hepatitis B virus infection, healthcare workers, vaccination coverage, pre-vaccination immunity screening, hepatitis B vaccination policy

## Abstract

**Background:**

Chronic infection with hepatitis B virus (HBV) is a major public health concern in South Africa. Hepatitis B virus is a highly infectious blood-borne virus causing liver disease. Healthcare workers (HCWs) are at high risk of occupational exposure.

**Objectives:**

This study aimed to investigate HBV vaccination amongst HCWs at a tertiary academic hospital in Gauteng province, South Africa.

**Method:**

Self-administered questionnaires were used to collect data from 500 consecutively sampled HCWs. Data were analysed using Stata version 12.

**Results:**

A total of 460 HCWs participated in the study. Most were women (68.7%), < 40 years of age (66.9%) and worked for < 10 years (66.0%). Almost 50.0% were either doctors or medical students and 40.3% were nurses or student nurses. Most HCWs in the age group of < 30 years (79.4%) had received at least 1 dose of HB vaccine. Prevaccination immunity screening was conducted on 17.5% of the HCWs, and only 11.0% reported to be protected against HBV. About 49.0% of HCWs were fully vaccinated. Post-vaccination immunity testing was conducted on 15.1%, and 24.0% of HCWs paid for vaccinations. Nursing staff and those with > 10 years of work experience were 2.5 and 2.6 times more likely to be vaccinated, respectively. Cleaning staff were less likely to be vaccinated.

**Conclusion:**

Although not all HCWs were fully vaccinated, our study found a higher proportion of fully vaccinated HCWs than previously reported in Gauteng Province. It is recommended that HB vaccination be promoted and a local vaccination policy, aligned with the national policy, be developed and implemented for all HCWs at the tertiary academic hospital.

## Introduction

Despite the widespread availability of a highly effective and safe hepatitis B (HB) vaccine, liver disease caused by chronic infection with the HB virus (HBV) remains a major public health problem.^1^ The World Health Organization (WHO) estimated that globally, 296 million people had chronic HB infection in 2019, resulting in 820 000 deaths, mostly from cirrhosis and hepatocellular carcinoma.^1^ Over the past three decades, the prevalence rates of chronic HBV infections have fallen substantially throughout the world, and this is attributable to the universal introduction of infant HBV vaccination in about 189 countries by 2018.^2^ In spite of these interventions, the WHO African Region has the second highest HBV prevalence, with 6.1% of the population estimated to be HB surface antigen (HBsAg) positive.^1^ Hepatitis B virus is a highly infectious blood-borne virus compared to hepatitis C virus (HCV) and human immunodeficiency virus.^3^

Healthcare workers (HCWs) are at an increased risk of occupational exposure to HBV infections^1,4^ through percutaneous injuries involving needle pricks and sharps, *inter alia,*^5^ and these account for most common occupational exposures.^6^ Healthcare workers may also transmit the virus to their patients^7^ through unsafe medical injection practices and use of inadequately sterilised medical equipment.^8^ Hepatitis B virus exposure amongst children and babies from infected HCWs and perinatal transmission can have devastating outcomes, leading to chronic hepatitis in about 95% of infected children later in life.^1^ In a systematic review conducted in Africa, the pooled 12-month prevalence estimate of occupational exposure because of percutaneous injuries was 35.9%, and this ranged from 16.4% in Southern Africa to as high as 67.9% in the Northern Africa Region.^6^

Hepatitis B virus infection is considered to be highly endemic in South Africa, like other sub-Saharan countries.^9^ In Gauteng province, the prevalence of HBV infection amongst HCWs was reported to be high, ranging from 2.4% to 14.3%, and most were positive for HBsAg and/or HBV deoxyribonucleic acid (DNA).^10^ Vaccination against HBV and appropriate use of post-exposure prophylaxis are the primary interventions for the prevention of HBV infection amongst HCWs. Despite the widespread availability of the HB vaccine for HCWs, one study found that only 19.9% of HCWs in Gauteng province were fully vaccinated against HBV (i.e. having received three or more doses of the HB vaccine) and 18.6% were protected through natural infection.^11^

The aim of this study was to determine coverage and the factors associated with HBV vaccination amongst HCWs at the DGMAH and SMU complex.

## Methods

### Definition of key terms

A HCW in this study is an individual who delivers service to patients either directly (e.g. medical staff members, medical and nursing students and nursing staff members) or indirectly (i.e. laboratory staff members, laboratory students and cleaners).

### Study design and setting

This cross-sectional study was conducted at Dr George Mukhari Academic Hospital (DGMAH), a tertiary academic hospital affiliated with the Sefako Makgatho Health Sciences University (SMU) (previously MEDUNSA) in the Gauteng province of South Africa.

### Study population and sample size

The study population included all at-risk HCWs as described above. At the time of data collection, there were an estimated 465 medical staff, 1150 medical students, 1468 nursing staff, 244 nursing students, 200 laboratory staff, 20 laboratory students and 380 cleaning staff (pers. comm., Human Resources Department, DGMAH 2011). There were 3927 HCWs eligible to take part in this study and all were invited to participate. A sample size of 453 was calculated at 80.0% power and 95.0% confidence interval using Epi Info^TM^ version 7 (Centers for Disease Control and Prevention, United States). The previous findings by Burnett et al., where 67.9% of HCWs in Gauteng province had received at least one dose of HB vaccine, informed the calculation of the final sample size.^11^ The final sample size was increased to 500, considering the possibility of nonresponse and incomplete questionnaires.

### Data collection tool

A 28-item self-administered questionnaire was developed and revised after a pilot study was conducted on 20 HCWs at Odi District Hospital (a Gauteng province feeder hospital referring to DGMAH). Data from the pilot study were not included in the main study. The questionnaire had information on the demographic characteristics of the participants, including history of occupational exposures, HBV vaccination status including doses received, pre-vaccination and post-vaccination immunity screening, results of screening and payment for vaccination.

#### Recruitment strategy and data collection

The study was widely publicised within the university and hospital complex. On data collection days, HCWs were verbally invited to ward meetings facilitated by heads of departments (Family Medicine, Paediatrics, Emergency Medicine, Internal Medicine, Surgery and Obstetrics and Gynaecology) and nursing managers in charge of the wards. Consecutive consenting participants were included in the study until the required 500 HCWs were reached.

A collection box was placed in each participating ward for participants to place their completed questionnaires. Questionnaires were collected daily, to be captured immediately. Two trained research assistants captured the data independently on Microsoft Excel spreadsheets. Consensus was used to decide when a discrepancy was observed on the entered data.

#### Data analysis

Statistical analysis was carried out using Stata Version 12. At the univariate level, data were described using mean and standard deviations (s.d.) for continuous variables and proportions for categorical variables. Bivariate analysis was conducted using Pearson’s chi-squared test of independence (Pearson *χ*^2^) to assess any potential association of HBV vaccination with any of the independent categorical variables. Because of adequate sample size, a binary logistic regression model (using the stepwise approach at a cut-off point of 0.05) was performed to determine factors associated with HBV vaccination.^12^ A *p*-value of less than 0.05 was considered statistically significant.

### Ethical considerations

Ethical approval to conduct the study was obtained from the MEDUNSA Research Ethics Committee (ref. no. MREC/H/75/2010:PG). Permission to conduct the study at DGMAH was obtained from the hospital management. No personal identifiers were collected on the study documents. Participation was voluntary in that participants could withdraw from the study at any point in time should they wish to do so.

## Results

Out of the 500 questionnaires distributed, 460 were returned, constituting a response rate of 92.0%. The results are summarised in Table 1. The mean age of participants was 34 years (s.d.: 11.58), and the ages ranged from 18 to 64 years. Table 2, indicates that of the participants who were in the age group of < 30 years (*n* = 218), 79.4% were vaccinated (173/218). Most HCWs were women (68.7%), single (58.5%) and were black people (93.0%), and most of the participants were vaccinated. Medical students were the majority of HCWs in our study (32.2%), and most of them were vaccinated (87.4%). The Pearson *χ*^2^ showed no association between the above-mentioned variables and HBV vaccination.

**TABLE 1:** Summary statistics of participants’ ages.

Variable	Mean	Min	Max	s.d.	Median
Age	34.02	18	64	11.58	30

s.d., standard deviation; Min, minimum; Max, maximum.

**TABLE 2:** Demographic characteristics of healthcare workers.

Variables	Not vaccinated		Vaccinated		Total		*p*	OR
	*n*	Row (%)	*n*	Row (%)	Frequency (*n*)	Col (%)		
**Age groups**								
18–29 years	45	20.6	173	79.4	218	47.40	0.722	
30–39 years	18	20.0	72	80.0	90	19.60		
40–49 years	17	23.9	54	76.1	71	15.40		
50–59 years	13	20.3	51	79.7	64	13.90		
60 years +	2	28.6	5	71.4	7	1.52		
Missing data	0	0.0	10	100.0	10	2.17		
**Median age**								
Less than 30 years	47	20.3	185	79.7	232	50.40	0.238	
More than 30 years	48	22.0	170	78.0	218	47.40		
Missing data	0	10	100.0	10	2.20			
**Gender**								
Female	59	18.7	257	81.3	316	68.70		
Male	36	25.0	108	75.0	144	31.30		
**Marital status**								
Married	34	20.9	128	79.0	162	35.20	0.329	
Single	58	21.6	211	78.4	269	58.50		
Divorced	2	11.8	15	88.2	17	3.70		
Cohabiting	0	00.0	10	100.0	10	2.20		
Missing data	1	50.0	1	50.0	2	0.43		
**Ethnicity**								
Black person	88	20.5	341	79.5	429	93.30	0.53	
White person	2	14.3	12	85.7	14	3.00		
Indian person	4	30.8	9	69.2	13	2.80		
Mixed race person	0	00.0	4	100.0	4	0.87		
**Professional group**								
Medical staff	20	25.3	59	74.7	79	17.20		
Medical students	32	31.6	116	78.4	148	32.20		
Nursing staff	12	9.6	113	90.4	125	27.20	0.004	3.2
Nursing students	13	21.7	47	78.3	60	13.10		
Laboratory staff	4	16.0	21	84.0	25	5.70		
Laboratory students	0	00.0	3	100.0	3	0.70		
Cleaners	12	66.7	6	33.3	18	3.90	0.002	0.69
**Years of experience**								
Less than 10 years	65	21.5	237	78.5	303	66.00	0.472	
More than 10 years	29	18.6	127	81.4	156	34.00		

OR, odds ratio; Col, column.

**FIGURE 1:**
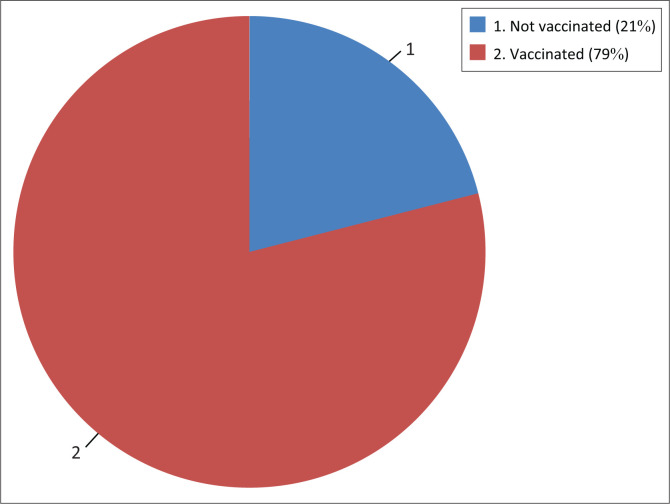
Percentage of healthcare workers who received at least one dose of hepatitis B virus vaccine (*n* = 460).

There was an association between work category or professional group and HBV vaccination, where nursing staff were three times more likely to be vaccinated (*p* = 0.004) than other professionals, and cleaners were less likely to be vaccinated (*p* = 0.02). The study showed that 66.0% of HCWs had less than 10 years of work experience, but the highest proportion of vaccinated individuals were amongst those with more than 10 years of work experience. However, no association was found (*p* = 0.472). Overall, the study showed that 79.3% of the participants were vaccinated with at least 1 dose of HB vaccine (Figure 1).

Table 3 shows that 48.8% of HCWs were vaccinated at DGMAH. Of all the HCWs who were vaccinated, 49.0% (*n* = 179) received three or more doses. The majority of the participants were tested neither before nor after vaccination. A small proportion of participants (11.0%) self-reported to have been protected against HBV. A total of 24.4% (89/365) reported to have self-funded their vaccination. Although the results are not included in the table, nursing students (34.8%, *n* = 31), medical students (33.7%, *n* = 30) and medical staff members (25.8%, *n* = 23) constituted the highest number of HCWs who reported to have paid for vaccination.

**TABLE 3:** Aspects of hepatitis B virus vaccination amongst healthcare workers at Dr George Mukhari Academic Hospital.

Variables	Not vaccinated		Vaccinated		Total	
	*n*	Col (%)	*n*	Col (%)	Frequency (*n*)	Col (%)
**Place of vaccination**						
Not vaccinated at DGMAH	0	0.0	171	46.9	171	37.2
Vaccinated at DGMAH	0	0.0	178	48.8	178	38.7
Missing	95	100.0	16	4.4	111	24.1
**Number of doses received**						
Don’t remember	0	0.0	39	10.7	39	8.5
1 dose	0	0.0	57	15.6	57	12.4
2 doses	0	0.0	90	24.7	90	19.6
3 doses	0	0.0	164	44.9	164	35.7
4 doses	0	0.0	15	4.1	15	3.3
Not vaccinated	95	100.0	0	0.0	95	20.1
**Tested before vaccination**						
No	0	100.0	272	74.5	272	59.1
Yes	0	0.0	64	17.5	64	13.9
Do not remember	0	0.0	13	3.6	13	2.8
Missing	95	85.6	16	4.4	111	24.1
**Tested after vaccination**						
No	0	0.0	276	75.6	276	60.0
Yes	0	0.0	55	15.1	55	12.0
Do not remember	0	0.0	12	3.3	12	2.6
Missing	95	81.2	22	6.0	117	25.4
**Protected against HBV**						
No	0	0.0	4	1.0	4	0.9
Yes	0	0.0	40	11.0	40	8.7
Do not remember	0	0.0	18	4.9	18	3.9
Missing	95	23.9	303	83.0	398	86.5
**Paid for vaccination**						
No	0	0.0	258	70.1	258	56.1
Yes	0	0.0	89	24.4	89	19.4
Not vaccinated	95	100.0	0	0.0	95	20.7
Do not remember	0	0.0	18	4.9	18	3.9

DGMAH, Dr George Mukhari Academic Hospital; HBV, hepatitis B virus.

Logistic regression analysis results (Table 4) show that the nursing staff members and those who have more than 10 years of work experience were 2.5 and 2.6 times more likely to be vaccinated with HB vaccine, respectively. The cleaning staff members, on the other hand, had an 85% less chance of being vaccinated, and the results are statistically significant.

**TABLE 4:** Factors associated with hepatitis B virus vaccination amongst health workers at Dr George Mukhari Academic Hospital.

Variables	Odds ratio	s.d.	*z*	*p*	95% confidence interval
Age	0.95945	0.02114	−1.88	0.060	0.91890; 1.00178
Gender	0.58741	0.22783	−1.37	0.170	0.27466; 1.25628
Marital status	1.51757	0.39709	1.59	0.111	0.90871; 2.53439
Ethnicity	0.89772	0.27787	−0.35	0.727	0.48941; 1.64669
Medical students	1.73570	0.97429	0.98	0.326	0.57766; 5.21525
Nursing staff	2.54692	1.20969	1.97	0.049*	1.00398; 6.46105
Nursing students	0.66836	0.34034	−0.79	0.429	0.24636; 1.81324
Lab staff	1.27605	0.75050	0.41	0.679	0.40294; 4.04109
Cleaners	0.14972	0.09487	−3	0.003*	0.04324; 0.51837
10+ years of exp	2.61223	1.25570	2	0.046*	1.01821; 6.70171

Note: Model parameters: Log likelihood = −142.97026, Pseudo *R*^2^ = 0.1204 and model *p*-value = 0.0000.

s.d., standard error.

* , *p* < 0.05.

## Discussion

### Overall vaccination coverage

This study found a high proportion of HCWs to have been vaccinated with at least one dose of HB vaccine. These figures are higher than that reported by previous studies conducted in South Africa. Two studies conducted in Gauteng province found that 53.4% and 67.9% of HCWs received at least one dose of the vaccine.^10,11^ Generally, HBV vaccination coverage amongst HCWs in developing countries has been found to be low. A Kenyan study found that 87.2% (483/554) of HCWs were not vaccinated,^13^ whilst two studies from Uganda found that 94.9% of 311^14^ and 93.8% of 370 HCWs^15^ were not vaccinated. In our study, the HBV vaccination coverage of 79.3% is comparable to results from most developed countries.^16,17,18,19^ This can be attributed to the fact that developed countries with low HBV endemicity have policies and regulations for the prevention of occupationally acquired nosocomial transmission of HBV, which includes vaccination of newly appointed HCWs.^20^ Healthcare workers should be vaccinated in the early stages of their careers.^21^ The National Department of Health recommends that HCWs should be vaccinated against HBV; however, vaccination is not mandatory in South Africa.^8,9,22^

Hepatitis B virus vaccination with at least one dose was highest amongst medical students and professional nurses in this study. These findings are similar to a previous study in Gauteng province reporting an 81.2% coverage amongst doctors and 65.3% and 67.6% amongst nursing students and professional nurses, respectively.^11^ In contrast, a study from Sweden found no association between professional category and vaccination uptake,^23^ whilst a study from Nigeria found that nurses were more likely to be vaccinated than doctors.^24^ A study conducted in the United States (US) showed that vaccination status was similar amongst medical (81.6%) and nursing (80.9%) staff members.^17^

### Testing before and after vaccination

Self-reported testing amongst HCWs before and after HBV vaccination was very low in this study. These low rates are comparable to rates in other studies conducted in Gauteng province, which found that only 24.5% of Tshwane metropolitan area nurses were tested before vaccination^25^ and that 27.6% of doctors and nurses in the Ekurhuleni metropolitan area were tested after vaccination.^26^ A slightly higher screening rate was observed in one Iranian study, where 56.8% of surgeons were screened for anti-HB level after vaccination.^18^ A study conducted in Saudi Arabia showed that of those vaccinated, 43.8% did not get antibody testing after vaccination whilst 48.2% of participants in the survey who had their antibody titre measured either did not know or did not check their results.^19^ A study in Kenya found that none of the vaccinated HCWs had been screened for anti-HBs.^13^ The low pre-immunisation screening numbers are not surprising in that the recent national guidelines for the management of viral hepatitis consider pre-immunisation screening to be unnecessary unless facilities find it cost-effective. Instead, post-immunisation screening is recommended after completion of the vaccination schedule.^8^

### Protection against hepatitis B virus

It is concerning that only 11.0% of vaccinated HCWs in the study reported to have been protected against HBV. The remainder of the HCWs either did not respond to the question or did not know if they were protected against HB. Most studies indicated little or no record of protection amongst HCWs against HBV, which might explain the observed paucity of data on their protection.^10^ A study conducted in South Africa amongst HCWs showed a low number of HBV-immune individuals (30.6%), with only 21.2% having a history of past immunisation against HBV.^22^ A study in Kenya showed that HCWs did not check the results for anti-HBs, which may result in HCWs believing that they are protected whereas they may have not responded to the primary vaccination course.^13^ This was the finding in a study from one industrialised country, which showed that 3.2% of vaccinated individuals had no measurable anti-HBs antibodies and required a revaccination programme.^27^ This low number could also be attributed to the fact that post-immunisation screening was never done at the time of data collection, and this become particularly important considering that up to 10.0% of adults who receive three HB vaccine doses do not develop protection.^8^ The policy also recommends routine post-immunisation testing, especially amongst health workers who are potentially at high risk of exposure.^8^ It remains clear that this was probably not a requirement even then, hence such low rates.

### Payment for vaccination

Only 24.4% of the HCWs in this study paid for their HBV vaccination, a number that may seem insignificant. This is more than in the study on Tshwane metropolitan area nurses, where only 4.8% reported to have paid for vaccination.^25^ Hepatitis B virus vaccination should be offered free of charge to HCWs by most health training institutions in Gauteng province; however, students were seemingly expected to be privately vaccinated at their own expense during the period of the study. A study in Pakistan and Ireland showed that HCWs would accept vaccination if it were free.^28,29^ Contrary to this finding, a study in the US found that vaccination coverage was low in hospitals with a policy that offered vaccination free of charge to HCWs.^17^ It is important to note that the HCWs in the US study did not have an identified risk of exposure, which may have influenced their opinion on the availability of free vaccination.

### Factors associated with hepatitis B virus vaccination

In terms of factors associated with the uptake of HBV vaccination, nurses were more likely than doctors to be vaccinated. These results are similar to another study which indicates that nurses were more likely to be vaccinated than doctors.^30^ Other studies, on the contrary, found that being a nurse was not associated with a lack of or incomplete vaccination,^31^ whilst another study found no association.^32^ Cleaning staff members have been reported to have a low coverage for HBV vaccination in this study, and this may be because of the lack of knowledge, awareness and educational training.^33,34^ The idea that cleaning staff members are less likely to be vaccinated is worrisome more especially for those performing tasks involving blood, contaminated bodily fluids and sharps. This could be a reflection that at the time of this study, they were inadvertently ignored. Cleaning staff belong to a category of workers who are recommended to receive routine pre-exposure HBV immunisation.^8^

Healthcare workers with more than 10 years of work experience were almost three times more likely to be vaccinated with HBV vaccine. These findings are similar to other studies which showed that newly recruited HCWs were likely to be unvaccinated or partially vaccinated compared with HCWs with more than 10 years’ work experience.^31,35^ This finding is contrary to a study conducted in Italy which showed that younger HCWs in Italy were more aware of being vaccinated and confirmed that young age and lowest years of employment were predictors of vaccine uptake.^16,21^ A study in Iran amongst surgeons also showed that younger surgeons were more likely to get vaccinated.^18^ Reports from industrialised countries show that despite repeated exposure to patients being positive for HBsAg, there is a decline in clinical HBV infection, which can be attributed to immunisation programmes.^19^

## Conclusion and recommendations

Occupational health and safety measures must be introduced at the DGMAH and SMU Complex to ensure that healthcare sciences students are vaccinated during their clinical training and receive routine post-immunisation testing in accordance with the national policy.^8,36^

Sufficient funding must be available in the budgeting process to ensure that all HCWs are fully vaccinated against HBV for free, and that they receive post-vaccination testing to confirm protection against HB. It has a potential to increase uptake.

All health workers with reasonable anticipated risk of exposure should be educated about the importance of vaccination against HBV, and systems should be in place in the healthcare facilities to ensure that HCWs are vaccinated early in their careers before exposure.^37,38^ It remains clear that further studies are warranted to assess progress since the introduction of the HBV policy in South Africa.

## Limitations

This study was conducted about a decade ago, prior to the introduction of national policy guidelines for the management of viral hepatitis in South Africa. The results, however, are somewhat reassuring, considering some of the practices that are in line with the current national guidelines in terms of vaccination and pre-immunisation screening. The findings of this study, however, may not be representative of the current state of affairs at the DGMAH. The study results also cannot be generalised to other settings as it was conducted in the DGMAH and SMU Complex only. Vaccination status was self-reported and not verified through vaccination records and therefore could be affected by recall bias of the participants.
